# Okra Flower Polysaccharide–Pea Protein Conjugates Stabilized Pickering Emulsion Enhances Apigenin Stability, Bioaccessibility, and Intestinal Absorption In Vitro

**DOI:** 10.3390/foods14111923

**Published:** 2025-05-28

**Authors:** Nuo Zhang, Jiale You, Xiaoli Yan, Hongchen Ji, Wenxuan Ji, Zhengyu Liu, Min Zhang, Peng Liu, Panpan Yue, Zain Ullah, Ting Zhao, Liuqing Yang

**Affiliations:** School of Chemistry and Chemical Engineering, Jiangsu University, Zhenjiang 212013, China; znuo3159@163.com (N.Z.); youjiale0220@163.com (J.Y.); 15249842090@163.com (X.Y.); jihongchen1997@gmail.com (H.J.); vincentttj@163.com (W.J.); liuzhengyuwd111@163.com (Z.L.); zhangmin@ujs.edu.cn (M.Z.); liupeng@ujs.edu.cn (P.L.); ypp1109@hotmail.com (P.Y.); zainullah@ujs.edu.cn (Z.U.)

**Keywords:** covalent conjugation, okra flower polysaccharide, emulsion stability, controlled release, bioavailability

## Abstract

The covalent interactions of polysaccharides and protein can improve the emulsification and stability of Pickering emulsions, which are promising systems for the delivery of active substances. Okra flowers, which commonly represent agricultural waste, have high-viscosity polysaccharides that can be used for the development of protein–polysaccharide-based emulsifiers. In this study, the Maillard reaction was performed under optimized conditions (70 °C, pH 10, and 12 h) with a 1:1 mass ratio to generate pea protein isolate (PPI)–okra flower polysaccharide (OP) conjugate with the highest grafting degree of 22.80 ± 0.26%. The covalent binding of OP facilitated variations in the secondary and tertiary structures of PPI, decreasing its particle size (from 535.70 to 212.05 nm) and zeta-potential (from −30.37 to −44.39 mV). The emulsifying stability of the emulsion stabilized by OP-PPI conjugates was significantly improved due to the formation of a stable interfacial layer, showing an 80.39% increase compared to that of free PPI. Simultaneously, the emulsions prepared with the conjugates demonstrated excellent stability across diverse environmental conditions by enhancing the interaction between the lipid and protein. Moreover, the conjugate-stabilized emulsion not only exhibited a higher encapsulation efficiency of 91.52 ± 0.75% and superior protective efficacy but also controlled the release of apigenin (API) during gastrointestinal digestion, achieving the highest API bioaccessibility (74.58 ± 1.19%). Furthermore, it also contributed to the absorption and transmembrane transport efficiency of API in Caco-2 cells, improving its bioavailability. These results confirmed that covalent conjugation with OP is a valuable strategy for enhancing the emulsifying features of PPI. The PPI–OP emulsion delivery system holds great potential for nutrient delivery.

## 1. Introduction

Pickering emulsions are formed by the strong adsorption of solid particles at the oil–water interface, driven by their excellent partial wettability in two phases [1]. Due to the irreversible adsorption and accumulation of particles at the oil–water interface, a closely packed layer can be formed, thus impeding the merging of emulsion droplets [2]. Therefore, Pickering emulsions exhibit excellent stability compared to traditional emulsions and show great potential in fields such as food, medicine, and cosmetics [3]. Emulsifiers are one of the most important components in emulsion formulations. Most conventional emulsions are stabilized by inorganic and synthetic polymer particles, which could cause toxicity and environmental problems [4]. For example, anionic surfactants may combine with proteins, enzymes, and phospholipid membranes, potentially bringing about biochemical alterations, including the modification of protein structure and malfunction of enzymes and phospholipid membranes [5]. Therefore, the development of nontoxic and biocompatible emulsions for the food industry remains an ongoing challenge. Over the last few years, biopolymers, including proteins and polysaccharides, have demonstrated the possibility of acting as Pickering stabilizers [6]. Insoluble particles (e.g., nanoparticles, micrometer-sized aggregates) formed by polysaccharides and/or proteins irreversibly adsorb at the oil–water interface through high-energy binding, stabilizing Pickering emulsion via the formation of a rigid physical barrier. In contrast to the classic emulsion stabilized by the soluble surfactants that rely on dynamic absorption and interfacial tension reduction, this particle-based stabilization mechanism confers superior resistance to environmental stress [1,7]. However, so far, the availability of food-grade stabilizers for Pickering emulsions remains limited.

Pea protein isolate (PPI) is a plant-based protein with high nutritional value, but its low surface charge and excessive aggregation significantly affect its emulsifying ability, thereby impeding its effectiveness as a Pickering stabilizer [8]. The current solution strategy, including electrostatic complexation and physical blending of proteins and polysaccharides, can help maintain the emulsifying properties of proteins [9]. Zhang et al. confirmed that non-covalent interactions with three anionic polysaccharides slowed down the aggregation of PPI emulsions, thus improving their stability [10]. Likewise, Aniya et al. used a combination of PPI and xanthan gum (XG) to emulsify mulberry anthocyanins, which significantly enhanced the thermal stability and the in vitro digestibility of anthocyanins [11]. However, non-covalent interactions as relatively weak interactions are unstable and are easily affected by environmental factors, including pH, salt ions, and temperature [12,13]. In addition, non-covalent complexes are more prone to dissociation risks during digestion and have limited protective efficiency for bioactive substances [14]. The Maillard reaction involves the reaction between an ε-amino group and a carbohydrate carbonyl group, facilitating the formation of complex conjugates with improved functional properties [15]. Previous studies have revealed that Maillard reaction conjugates exhibit enhanced emulsifying properties, solubility, and stability. There is also the potential for these conjugates to encapsulate hydrophobic bioactive substances within Pickering emulsions [16]. In our previous research, okra flower polysaccharides (OP) were isolated from *Abelmoschus esculentus* (okra) flowers, a waste product of okra cultivation. These polysaccharides exhibited high viscosity, good stability, non-toxicity, and a high biocompatibility [17]. These features may help to enhance the mechanical strength of the emulsion interface film and the tolerance of emulsions to environmental stress, thereby improving the emulsification stability. Additionally, they may promote the interaction between conjugates and the intestinal mucus layer, enhancing the absorption and transport of hydrophobic compounds. Hence, we speculate that the covalent conjugation of plant proteins with OP may provide a breakthrough for food-based stabilizers, combining robust interfacial engineering with targeted bioaccessibility enhancement.

Apigenin possesses diverse biological activities, such as antioxidant, anti-inflammatory, anti-cancer, and neuroprotective effects [18]. Its biological activity mainly depends on its bioavailability after digestion and absorption. However, its low solubility and poor bioavailability in the human body limit its application in the modern industry [19,20]. Therefore, this study aimed to study the potential of PPI–OP conjugates synthesized by the Maillard reaction under wet-heating conditions serving as Pickering stabilizers in food-related applications. Firstly, based on the fact that the extent of the Maillard reaction would affect the conjugates’ features, synthesis conditions were optimized. Then, physicochemical and functional properties, morphology, and mechanisms of covalent conjugation were examined and contrasted with those of free PPI and PPI–OP mixture. Afterwards, a Pickering emulsion stabilized by the PPI–OP conjugate was developed to encapsulate apigenin. The emulsions’ storage stability, microstructure, rheological properties, and formation mechanism were investigated. Finally, apigenin (API), as a model bioactive substance, was encapsulated within the emulsions stabilized with PPI–OP conjugate, and the encapsulation efficiency as well as in vitro release rate was examined to verify its stability, controlled-release performance, and bioaccessibility. An in vitro model of Caco-2 cells was used to determine the cellular uptake and transmembrane transport efficiency of apigenin in diverse emulsions. This study aims to provide new insights into the use of Maillard covalent bonding to enhance the interaction between PPI and OP for the preparation of stable emulsions and to contribute to the expansion of PPI–OP emulsions in the protection and delivery of Apigenin.

## 2. Materials and Methods

### 2.1. Chemical and Reagents

Dried okra flower (Hunan Qinshi Pharmaceutical Co., Ltd., Changsha, Hunan, China). Pea protein isolate (PPI, protein~80%) (Yuanye Biotechnology Co., Ltd., Shanghai, China). Soybean oil (Yihai Kerry Co., Ltd., Shanghai, China). Pepsin (P6887), pancreatin (P7545), and lipase (L3126) were supplied by Sigma-Aldrich Company (Steinheim, Germany). Apigenin (API) with a purity greater than 98% (HPLC) was obtained from Macklin Biochemical Technology Co., Ltd. (Shanghai, China). The malondialdehyde (MDA) assay kit (A003-1) was purchased from Nanjing Jiancheng Biotechnology Co., Ltd. (Nanjing, Jiangsu, China). All other chemicals used in this study were of analytical grade.

### 2.2. Preparation of Okra Flower Polysaccharides

The frozen okra flowers were crushed and sieved through an 80-mesh sieve. The resulting powder was mixed with deionized water at a solid–liquid ratio of 1:30. The pH was adjusted to 2.0 using HCl (1.0 M) and heated at 70 °C for 3 h, and afterwards it was centrifuged at 8000 rpm for 15 min. The concentrated supernatant was precipitated with 60% ethanol and left overnight at 4 °C. The precipitates were obtained by centrifugation at 8000 rpm for 15 min. After redissolving, the solution was dialyzed against distilled water using a dialysis bag with a molecular weight cut-off (MWCO) of 8000–14,000 Da for 48 h. Finally, the okra flower polysaccharide (OP) was obtained by freeze-drying for 2 days. The monosaccharide composition and molecular weight of OP were determined as described by Zhang et al. [17]. In brief, 10 mg of OP was mixed with 5 mL of 2 M trifluoroacetic acid (TFA). Hydrolysis was carried out at 100 °C for 4 h in a sealed reactor under a nitrogen atmosphere. After the hydrolysis process, 0.5 M of PMP methanol solution and 0.3 M of NaOH solution were added to the hydrolysate. The mixture was incubated at 70 °C for 40 min, followed by neutralization with a 0.3 M HCl solution. After CH_3_Cl extraction, the sample in the aqueous layer was used for high-performance liquid chromatography (HPLC) analysis. The molecular mass was analyzed using HPSEC integrated with multi-angle laser-light scattering (MALLS, DAWN HELEOS II, λ = 658 nm; Wyatt Technologies Corporation, Santa Barbara, CA, USA). The polysaccharide was dissolved in 0.1 M NaCl and filtered through 0.45 μm polytetrafluoroethylene membranes before injection into the system. The structure characterization showed that OP was a heteropolysaccharide with an average molecular weight (Mw) of 2.761 × 10^6^ Da, composed of Rha, GlcA, GalA, Glc, Gal, Xyl, and Ara with a molar ratio of 1.547:0.322:4.012:41.020:25.504:7.128:13.321.

### 2.3. Preparation of PPI–OP Maillard Conjugates

The reaction conditions were optimized based on the results of the pre-experiments (the results are shown in Supplementary Material). A 1% (*w*/*v*) pea protein isolate (PPI) and 1% (*w*/*v*) okra flower polysaccharide (OP) solutions were mixed at a 1:1 (*v*/*v*) ratio. The pH was adjusted to 10.0 using NaOH (1.0 M). The mixture was incubated at 70 °C for 12 h. After thermal treatment, the reaction was terminated by cooling the solutions in an ice bath for 5 min. Each solution was then subjected to dialysis at 4 °C for 24 h to obtain PPI–OP Maillard conjugates. A portion of each dialyzed solution was preserved by adding 2.5 mg of sodium azide (NaN_3_) (0.025%, *w*/*v*) and stored at 4 °C. The remaining portion was freeze-dried for further analysis. Under these reaction conditions, the grafting degree of the PPI–OP conjugate was 22.80 ± 0.26%.

### 2.4. Determination of Grafting of PPI–OP Conjugates

The grafting degree (DG) of the PPI–OP conjugates was evaluated by measuring the reduction in free amino groups after the Maillard reaction using the o-phthaldialdehyde (OPA) method as described [21]. Briefly, a 200 μL sample solution containing 2.5 mg/mL of protein was mixed with 4 mL of freshly prepared OPA reagent. The reaction mixture was incubated in a water bath at 35 °C for 2 min, and then the absorbance was measured immediately at 340 nm. Three independent experiments (*n* = 3) were conducted with triplicate replicates. The DG were calculated using the following formula:

(1) $$DG=\frac{A_{1}-A_{2}}{A_{1}}\times 100$$

where A_1_ represents the content of free amino groups in the PPI–OP mixture, and A_2_ represents the content of free amino groups in the covalent complex.

### 2.5. The Structure Features of PPI–OP Conjugates

#### 2.5.1. Fourier Transform Infrared Spectroscopy (FTIR)

Freeze-dried samples were thoroughly ground with anhydrous potassium bromide at a ratio of 1:100 (*w*/*w*). The FTIR spectra of the PPI, OP, and PPI–OP mixtures and conjugates were recorded using an IR5 FTIR spectrometer (Techcomp Scientific Instruments Co., Ltd., Shanghai, China) with a scanning range of 4000–500 cm^−1^. Three independent experiments (*n* = 3) were conducted.

#### 2.5.2. Circular Dichroism (CD)

The CD spectroscopy of PPI, OP, and PPI–OP mixtures and conjugates were recorded using a spectropolarimeter (Model JASCOJ-815, JASCO Corporation, Tokyo, Japan), within a scan range of 190–260 nm. All samples were diluted with distilled water to 0.2 mg/mL. The CD spectra were analyzed using DICHROWEB (Birkbeck Research Group, University of London, Malet St, Bloomsbury, UK) to calculate the secondary structure content. Three independent experiments (*n* = 3) were conducted with triplicate replicates.

#### 2.5.3. Intrinsic Fluorescence

The samples were diluted to 1 mg/mL with 0.1 mol/L PBS (pH 7.4) solution. The fluorescence intensity of the PPI, OP, and PPI–OP mixtures and conjugates were recorded using an FL970 fluorescence spectrophotometer (Techcomp Scientific Instruments Co., Ltd., Shanghai, China). During the measurement, the excitation wavelength (λ_ex_) was set at 280 nm, and the emission wavelength (λ_em_) was scanned across the range of 300–500 nm (slit = 5 nm). Three independent experiments (*n* = 3) were conducted with triplicate replicates.

#### 2.5.4. Particle Size and Zeta-Potential

A laser diffraction particle size analyzer (Mastersizer3000, Malvern Instruments, Worcestershire, UK) was used to determine the particle size and distribution. The ζ-potential was characterized by a nano laser particle size instrument (ZEN3700, Malvern Instruments, Worcestershire, UK), and the samples were diluted to 0.1 mg/mL with distilled water. Three independent experiments (*n* = 3) were conducted with triplicate replicates.

#### 2.5.5. Turbidity

The turbidity values were measured using a TU1800 ultraviolet-visible photometer (Puxi General Instrument Co., Ltd., Beijing, China) at a wavelength of 600 nm. Each sample was measured in triplicate. Three independent experiments (*n* = 3) were conducted with triplicate replicates.

#### 2.5.6. Scanning Electron Microscopy (SEM)

The PPI, OP, and PPI–OP mixtures and conjugates were attached to a conductive paste and plated with gold in an E-1010 ion sputter. The morphology of each sample was observed and photographed using a scanning electron microscope (JSM-7610F PLUS, JEOL Ltd., Tokyo, Japan). Three independent experiments (*n* = 3) were conducted.

### 2.6. Preparation of Emulsion

The PPI–OP conjugate, PPI–OP mixture, OP, and PPI were individually dissolved in distilled water to achieve a final concentration of 2 mg/mL. Subsequently, 17 mL of each solution was mixed with 3 mL of soybean oil (oil phase fraction: 15% *v*/*v*) and homogenized at 12,000 rpm for 2 min using a high-speed disperser (T18, IKA-Werk, Staufen, Germany).

### 2.7. Characterization of PPI–OP Conjugate-Stabilized Pickering Emulsions

#### 2.7.1. Emulsifying Property

Emulsification activity index (EAI) and emulsification stability index (ESI) were evaluated according to the method described in the previous study [22], with slight modifications. After emulsion preparation, 50 μL of emulsions were collected at 0 and 10 min, respectively, and were immediately mixed with 4.95 mL of 0.1% (*w*/*v*) SDS solution. Then, absorbances of mixtures were recorded at 500 nm. Three independent experiments (*n* = 3) were conducted with triplicate replicates. EAI and ESI were calculated using the following formulas:

(2) $$EAI(m^{2}/g)=\frac{2\times 2.303\times A_{0}\times N}{(1-\varphi )\times {c\times 10}^{4}}$$

(3) $$ESI(\%)=\frac{A_{10}}{A_{0}}\times 100$$

where EAI is the emulsification area of 1 g of protein; A_0_ is the absorbance of emulsions at 0 min; N is the dilution factor; c is the concentration (g/mL); φ is the volume of the oil phase (0.15); and A_10_ is the absorbance after standing for 10 min.

#### 2.7.2. Microstructure Observation of Pickering Emulsions

##### Optical Microscope

An amount of 20 μL of fresh emulsion was added on a glass slide. After being covered with a cover slip, the microstructure was immediately observed under a 40× objective lens using a microscope (DM750, Leica Microsystems, Wetzlar, Germany).

##### Confocal Laser Scanning Microscope (CLSM)

The protein and soybean oil in the emulsions were stained with Nile blue (0.1% wt) and Nile red (0.1% wt), respectively. Then, a 15 μL sample was placed on a slide gently covered with 0.17 mm coverslips and imaged at excitation wavelengths of 488 nm and 640 nm.

#### 2.7.3. Emulsion Stability Evaluation

The stability of emulsions under environmental stress was evaluated, including different pH values (3, 5, 7, 9, and 11), ionic concentrations (0, 100, 300, and 500 mmol/L), and lipid oxidation conditions. The average particle size and zeta potential were measured using dynamic light scattering (DLS) analysis. Lipid hydroperoxide products in the emulsions were determined by the peroxide value (POV) on days 0, 1, 2, 3, 4, 6, and 8. Moreover, the content of malondialdehyde (MDA) was measured by an assay kit to evaluate the secondary oxidation products. Three independent experiments (*n* = 3) were conducted with triplicate replicates.

#### 2.7.4. Raman Spectra Analysis

An amount of 1 mL of the emulsion was placed on a 35 mm circular quartz dish. Under excitation with a 532 nm laser, Raman spectra were obtained using a Raman spectrometer (DXR, Thermo Fisher Scientific, Waltham, MA, USA) with a He-Ne detector. Spectra were recorded from 3300 to 100 cm^−1^, with a 40 s integration time. Laser power was adjusted to 10 mW using an optical meter. Three independent experiments (*n* = 3) were conducted.

#### 2.7.5. Rheological Characterization

The viscoelastic characteristics of the emulsions were determined by means of a shear rheometer (DHR-3, TA Instruments, New Castle, DE, USA). Measurements were carried out at 25 °C using a parallel plate geometry (40 mm diameter) with a 1 mm gap. The apparent shear viscosity of the emulsions was measured over a shear rate range of 0.1–500 s^−1^. An oscillating frequency sweep was conducted with a constant 1% applied strain (in the linear viscoelastic region) from 0.1 to 10 Hz. Three independent experiments (*n* = 3) were conducted with triplicate replicates.

### 2.8. Preparation of Pickering Emulsions Loaded with Apigenin (API)

An appropriate amount of API was dissolved in soybean oil and magnetically stirred at a rotation speed of 500 rpm for 3 h to obtain an oil phase with 0.1% wt (*w*/*v*) API. The preparation process of the API-loaded emulsion was identical to that of the above emulsion. The emulsions stabilized by PPI, the PPI–OP mixture, and the PPI–OP conjugate were named as PPI-API, Mixtures-API, and Conjugates-API emulsion, respectively.

The emulsion embedded with API was added to an anhydrous ethanol solution at a ratio of 1:9 (*v*/*v*) and mixed thoroughly, followed by centrifugation to obtain the supernatant for analysis. This process was repeated three times, and the absorbance of the ethanol solution was recorded at 340 nm. Three independent experiments (*n* = 3) were conducted with triplicate replicates. The encapsulation efficiency (EE) of API was calculated as follows (4):

(4) $$EE(\%)=\frac{m_{1}}{m_{2}}\times 100$$

where m_1_ and m_2_ are the content of encapsulated and total API in emulsions, respectively.

### 2.9. Stability Determination of API in Emulsion

An amount of 20 mL of PPI-API emulsions, Mixtures-API emulsions, and Conjugates-API emulsions were individually placed in a transparent glass bottle. All of them were stored at 25 °C for 0–7 days. Another batch of emulsions with the same volume were stored at 95 °C for 1–5 h. The third batch of emulsions with the same volume was exposed to ultraviolet irradiation for 5 h. The contents of API in the various emulsions were determined daily or hourly using the method in Section 2.8. Three independent experiments (*n* = 3) were conducted with triplicate replicates. The retention rate of API was calculated using the following formula:

(5) $$API\;retention\;rate(\%)=\frac{C\times V}{M}\times 100$$

where C is the concentration of API released by the emulsion (mg/mL), V is the volume of the emulsion (mL), and M is the amount of API in the initial emulsion (mg).

### 2.10. In Vitro Digestion, Absorption, and Transport Investigation

#### 2.10.1. Controlled Release

The gastrointestinal stability of apigenin was evaluated using an in vitro digestion model, based on the international network INFOGEST protocol [23]. The digestive parameters are shown in Table S1. Three independent experiments (*n* = 3) were conducted with triplicate replicates.

Gastric phase: The simulated gastric fluid (SGF) contained 47.2 mM of sodium chloride and 2000 U/mL of pepsin. Amounts of 20 mL of apigenin-loaded PPI emulsions, PPI–OP mixture emulsions, and PPI–OP conjugate emulsions were individually mixed with SGF solution at a ratio of 1:1 (*v*/*v*). The pH of the mixtures was adjusted to 3.0, and then the mixtures were incubated in a constant-temperature shaker (37 °C, 100 rpm) for 120 min to simulate in vitro gastric digestion.

Small intestinal phase: The gastric digestion sample was mixed 1:1 (*v*/*v*) with SIF containing 2000 U/mL of lipase, 100 U/mL of pancreatin, and 10 of mM bile salt. The pH was adjusted to 7.0 by adding NaOH solution, and the digestion samples were incubated at 37 °C in a shaking water bath for 180 min.

Upon completion of in vitro digestion, the resultant emulsion digesta was centrifuged (12,000 rpm, 10 min). The micellar phase was obtained and vortexed with absolute ethanol at a 1:10 (*v*/*v*) ratio, followed by centrifugation (4500 rpm, 15 min). Finally, the supernatant was gathered, and its absorbance was determined at 340 nm. Three independent experiments (*n* = 3) were conducted with triplicate replicates. The bioaccessibility of API was calculated with the following Formula (6):

(6) $$Bioaccessibility(\%)=\frac{C_{Digesta}}{C_{Total}}\times 100$$

where C_Digesta_ is the concentration of apigenin in the digested fluid from the small intestine, and C_Total_ is the concentration of apigenin in the emulsion.

#### 2.10.2. Caco-2 Cell Viability Assay

To determine the optimal concentration of digesta, the Cell Counting assay Kit-8 (CCK-8) was used to measure the viability of Caco-2 cells (purchased from the Shanghai Cell Bank of the Chinese Academy of Sciences, Shanghai, China). Cells in the logarithmic growth phase were seeded into a sterile 96-well plate at a density of 1 × 10^5^ cells/mL, and 100 μL digesta, diluted at ratios of 1:5, 1:10, 1:20, 1:30, 1:40, 1:50, 1:60, 1:70, 1:80, 1:90, and 1:100, was added to each well. The plate was incubated at 37 °C with 5% CO_2_ for 24 h. The supernatant was then discarded, and the wells were rinsed twice with sterile PBS. Subsequently, 100 μL of culture medium containing 10% CCK-8 solution was added to each well, and the plate was incubated at 37 °C with 5% CO_2_ for 1 h. The absorbance of each well was measured at 450 nm. Three independent experiments (*n* = 3) were conducted with triplicate replicates. The cell viability was calculated using the following formula:

(7) $$Cell\;viability\left(\%\right)=\frac{A_{s}-A_{b}}{A_{c}-A_{b}}\times 100$$

where A_s_ is the absorbance of digesta, A_b_ represents the absorbance of the blank group without cells or digesta, and A_c_ is the absorbance of the control group without digesta.

#### 2.10.3. Establishment of the Caco-2 Cell Absorption Model

When cell confluence reached 80%, the cells were resuspended in DMEM medium containing 20% fetal bovine serum. The cells were then seeded into a 12-well Transwell culture plate (Corning Incorporated, Corning, NY, USA), with a polycarbonate membrane insert (diameter: 12 mm, and pore size: 0.4 μm) at a density of 1 × 10^5^ cells/mL. The apical side (AP side) and basolateral side (BP side) were filled with 0.4 mL and 0.6 mL of the cell suspension, respectively. The cells were incubated at 37 °C with 5% CO_2_. The medium was changed every other day. During this period, the transepithelial electrical resistance (TEER) value of the Caco-2 cell monolayer was measured using a voltohmmeter (Millicell-ERS, Millipore Corporation, Billerica, MA, USA) until an intact monolayer formed. After approximately 22 days of culture, cells with a TEER value exceeding 500 Ω·cm^2^ were selected for further experiments. Moreover, the activity on the AP side of the cell monolayer was significantly higher than that on the BL side, which indicated that the cells had differentiated into a polarized state with the necessary carriers and enzymes for absorption and transport.

#### 2.10.4. Transport and Uptake of API by Caco-2 Cells

A 22-day-cultured Caco-2 cell monolayer was equilibrated with Hanks’ balanced salt solution (HBSS) at 37 °C in an incubator for 30 min. After discarding the buffer solution, the model was rinsed twice with pre-warmed HBSS at 37 °C. Afterwards, 0.5 mL of intestinal juices was added to the apical (AP) side of the cells, while 1.5 mL of blank HBSS was added to the basolateral (BP) side as the receiving solution. The transport culture tanks were incubated in a constant temperature shaking incubator (37 °C, 50 rpm). After 120 min, samples were taken from the BP side, and the API concentration was measured at 340 nm. Three independent experiments (*n* = 3) were conducted with triplicate replicates. The transport rates were calculated using Formula (8). Finally, the treated cells were gently scraped off using a cell scraper and transferred to EP tubes. The collected cells were lysed by sonication and centrifuged. The content of API in the supernatant was measured to evaluate the uptake of API. The bioavailability of API was calculated using the following formulas:

(8) $$Transport\;rate(\%)=\frac{m_{BL}}{m_{AL}}\times 100$$

(9) $$Bioavailablity\left(\%\right)=\frac{m_{uptake}}{m_{digesta}}\times 100$$

where m_BL_ is the amount of API transported on the basolateral side, and m_AL_ represents the mass of API added to the apical side. m_uptake_ is the amount of API absorbed by the cells, and m_digesta_ indicates the content of API from the intestinal juices.

### 2.11. Statistical Analysis

The results are presented as mean values with their standard deviations. Duncan’s multiple range test and ANOVA were employed to identify significant differences using SPSS 19.0, with the significance level set at *p* < 0.05. Graphs were generated using Origin 2024 software.

## 3. Results and Discussion

### 3.1. Features of the Structure of PPI–OP Conjugates

#### 3.1.1. FTIR Analysis

Infrared spectroscopy served to determine the structural alterations of conjugates during the Maillard reaction. OP had the typical absorption peaks of common polysaccharides. The largest peak in the range of 3600–3200 cm^−1^ corresponded to the stretching vibration of –OH. Meanwhile, the peaks at 1609 and 1384 cm^−1^ were associated with the asymmetric and symmetric stretching of the C=O group in the COO– of galacturonic acid, respectively. The –CH absorption vibration in the range of 1000–1200 cm^−1^ suggested the existence of pyranose rings in OP [17]. In the spectrum of PPI, the characteristic bands were observed, including amide I (1653 cm^−1^ corresponding to C=O stretching), amide II (1545 cm^−1^ relating to N–H deformation), and amide III (1395 and 1242 cm^−1^ associated with C–N stretching and N–H bending vibrations, respectively) [15]. The 2950 cm^−1^ absorption band was attributed to the antisymmetric stretching of C–H within CH_2_ and CH_3_ groups [24]. The spectrum of the PPI–OP mixtures appeared as a superposition of the spectra of pure protein and the polysaccharides. The basic peptide bond structure and the broad absorption peak of the hydroxyl stretching vibration were still present. However, in the PPI–OP conjugates, there was a reduction in the absorption peak intensity of the amide I band of pea protein. Similarly, the intensities of characteristic absorption peaks of polysaccharides, such as the C–O–C stretching vibration peak at 1000–1100 cm^−1^, also differed in the PPI–OP mixtures compared to pure polysaccharides. This may be due to the strong interaction between the protein and the polysaccharides, which affected the vibration intensity of the amide bond and altered the conformation and aggregation state of the polysaccharide molecular chain, leading to changes in the absorption peak intensities. The covalent grafting between PPI and OP resulted in the disappearance of the NH_2_ energy band and the emergence of new energy bands corresponding to Maillard reaction products [21]. Compared to free PPI, the absorption band at 1545 cm^−1^ in the conjugate was significantly reduced, which indicated that the consumption of some functional groups, including NH_2_, especially from lysine, may have participated in the Maillard reaction [25]. In addition, the amide I wavenumber shifted from 1653 cm^−1^ to 1640 cm^−1^, with an increase in absorption intensity, which was attributed to the C≡N stretching vibration of Schiff base products during the Maillard reaction [26,27]. The wavenumber shifts from 1439 cm^−1^ to 1451 cm^−1^ suggested that hydrogen bonds might have been disrupted during the conjugation process. In addition, several new peaks also appeared in the conjugates in the wavenumber range of 1231 to 1451 cm^−1^, indicating the formation of amide bonds between the -COOH of OP and the -NH_2_ of PPI [27]. These observations further validated the formation of the PPI–OP conjugate.

#### 3.1.2. CD Analysis

**Figure 1:**
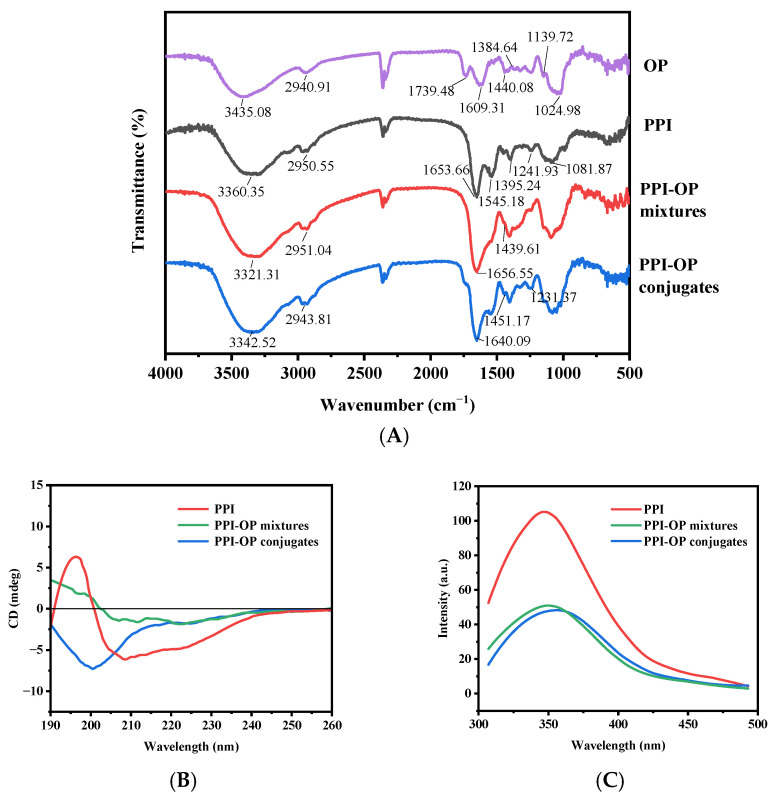
Fourier transform infrared spectroscopy (**A**); the circular dichroism spectrum (**B**) and the intrinsic fluorescence spectrum (**C**) of OP: okra polysaccharide; PPI: Pea protein isolate; PPI–OP mixtures; PPI–OP conjugates.

Subsequently, circular dichroism (CD) spectral analysis was conducted to assess the alterations in the secondary structure of PPI after conjugation [26]. The CD spectrum of PPI exhibited a positive peak at 196 nm and a negative peak at 209 nm (Figure 1), consistent with the previous literature [28]. The negative peaks at 201 nm and 208–222 nm corresponded to disordered random coil structures and ordered α-helix and β-sheet structures, respectively. Compared with PPI, the negative peaks of the PPI–OP mixtures and conjugates at 208–222 nm were weakened, which indicated a decrease in the corresponding α-helix content from 26.2 ± 0.04% to 17.0 ± 0.03% and 11.9 ± 0.04% and a reduction in β-sheet content from 31.3 ± 0.11% to 29.1 ± 0.12% and 27.8 ± 0.10%, respectively (Table 1). The peak intensity of the PPI–OP conjugates at 201 nm increased significantly, corresponding to an increase in the random coil content from 32.1 ± 0.09% to 43.6 ± 0.12%. The incorporation of OP modified the CD profile of PPI, which indicated that the Maillard reaction during the heat treatment influenced its secondary structure. Studies have shown that ordered secondary structures are usually buried within the polypeptide chains. Therefore, the decrease in structures verified that the conjugation of polysaccharides transformed the spatial conformation of proteins, leading to a looser protein structure, and implied an enhanced flexibility of protein molecules [26]. Wen et al. also reported similar results that the contents of α-helix and β-sheet in the SPI-lentinan conjugates were lower than those in the original SPI, while the β-turn and random coil contents were higher [21]. Similarly, Zang et al. suggested that an increase in the random coil structures is highly beneficial for enhancing the emulsifying properties of proteins. Hence, they are able to adsorb onto the surface of oil droplets more effectively [29].

#### 3.1.3. Intrinsic Fluorescence Analysis

Intrinsic fluorescence spectra serve as a valid approach for identifying the tertiary structure of proteins and are chiefly employed to detect alterations in the polarity of the microenvironment encircling hydrophobic amino acid residues [30]. In this study, fluorescence spectra of PPI, OP, PPI–OP mixtures, and PPI–OP conjugates are shown in Figure 1. The findings indicated that the peak emission wavelength (λ_max_) of PPI was roughly 347 nm, which was characteristic of tryptophan fluorescence. In comparison, the λ_max_ of the PPI–OP mixture remained almost unchanged at 347 nm [30,31]. However, the λ_max_ of PPI–OP conjugates exhibited a red-shift from 347 nm to 356 nm. Previous studies have suggested that the exposure of more Trp residues to a hydrophilic environment due to the partial or complete unfolding of the tertiary structure of proteins results in a more polar microenvironment around the chromophores, thereby increasing λ_max_ [32]. The results in this study suggested that the unfolding of the protein structure subsequent to the covalent grafting of PPI with OP caused tryptophan residues in PPI to orient toward a more hydrophilic environment. Additionally, the fluorescence intensity of the PPI–OP conjugates was significantly lower than that of the free PPI. High fluorescence intensity is typically associated with hydrophobic amino acid residues like tryptophan, tyrosine, and phenylalanine situated in the folded core region of the protein, forming a relatively stable hydrophobic environment [33]. The observed decrease in fluorescence intensity can be ascribed to the unfolding of the protein structure after OP was covalently grafted with PPI, which may result in the re-aggregation of hydrophobic groups through hydrophobic interactions. Furthermore, the chemical modification of proteins may also change the chemical groups surrounding the tryptophan residues to interfere with the electronic structure, thus impeding its fluorescence emission [34]. The covalent grafting of OP to PPI served to shield the inherent fluorescent amino acids, which also contributed to fluorescence quenching. In conclusion, the covalent attachment of OP to PPI augmented the fluorescence quenching and markedly modified the tertiary structure of PPI, thereby validating the success of the covalent attachment process.

#### 3.1.4. Turbidity and DLS Analysis

Turbidity reflects the degree of particle aggregation in a solution. As shown in Table 2, the turbidity of the PPI–OP mixtures slightly decreased, indicating that the addition of OP improved protein self-aggregation through the interaction between OP and PPI during the mixing process. The turbidity of PPI–OP conjugates was significantly reduced compared to PPI and PPI–OP mixtures, which revealed that covalent bonding disrupted the original interactions between protein molecules, resulting in a decrease in protein–protein molecular aggregates. Furthermore, the significant reduction in particle size indicated that the structure of the complexes became more compact, suggesting a strong binding interaction between PPI and OP. Smaller conjugates are more likely to be adsorbed onto the interface, providing a theoretical basis for improving emulsifying activity [35]. The absolute potential values of the complexes were increased, especially in PPI–OP conjugates, reaching 44.39 ± 1.53 mV. This indicated that the interaction between OP and PPI improved the stability of PPI nanoparticles by altering the surface charge distribution and enhancing the electrostatic repulsion between particles, further improving their stability in emulsified systems [36].

#### 3.1.5. Morphology Analysis

**Figure 2:**
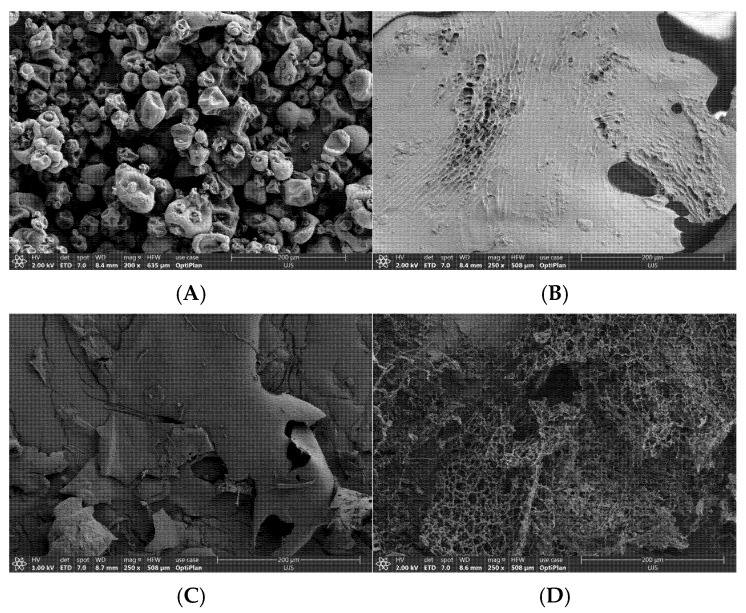
The SEM images of (**A**) PPI; (**B**) OP; (**C**) PPI–OP mixtures; and (**D**) PPI–OP conjugates.

The solubility of biopolymers could be significantly affected by their interfacial and morphological characteristics. Therefore, the surface morphology of PPI–OP conjugates was analyzed using JSM-7610F Scanning Electron Microscopy (JEOL Ltd., Tokyo, Japan). The morphology of PPI primarily exhibited a spherical structure (Figure 2A), whereas lyophilized OP displayed an irregular, broken-layered structure with a continuous honeycomb-like porous interior (Figure 2B), which is a typical feature of lyophilized powders [24]. In terms of the PPI–OP mixtures, the coexistence of spherical and flaky shapes indicated a physical mixing matrix of the two biopolymers (Figure 2C). In comparison, the morphology of PPI–OP conjugates (Figure 2D) differed from that of free PPI and PPI–OP mixtures, forming a non-homogeneous hybrid network structure. After the close adhesion of OP to the surface of PPI, the conjugate’s surface became rougher and formed a multi-porous structure. The pores exhibited varying sizes and distributions, indicating a non-uniform morphology. This observation indicated that PPI and OP were tightly interconnected through the glycosylation [37]. Comparable research has been recorded in previous studies where SEM morphology revealed comparable changes after corn fiber gum conjugated with milk protein [38]. Additionally, such a surface morphology can promote the interactions between the conjugates and water, thereby increasing the solubility of PPI [2].

### 3.2. Emulsifying Properties

**Figure 3:**
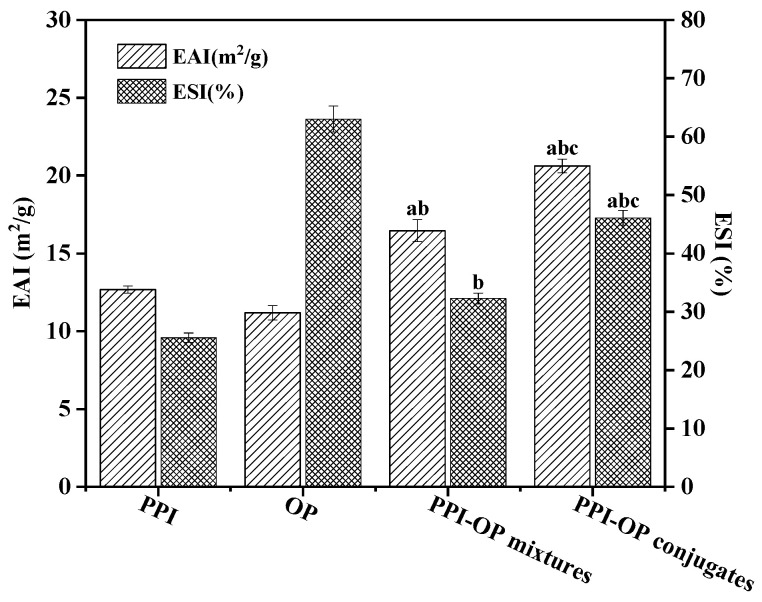
Emulsification activity index (EAI) and emulsification stability index (ESI) of PPI, OP, PPI–OP mixtures, and PPI–OP conjugates. “a”, “b”, and “c” represent a significant difference (*p* < 0.05) vs. the PPI group, the OP group, and the PPI–OP mixtures group, respectively.

To evaluate the emulsification performance of the emulsifiers, the EAI and ESI of emulsion stabilized by PPI, OP, PPI–OP mixtures, and PPI–OP conjugates were determined. As shown in Figure 3, the EAI and ESI of free PPI were 12.68 m^2^/g and 25.55%, respectively. Compared to free PPI, the EAI and ESI values of PPI–OP mixtures were slightly enhanced, reaching 16.46 m^2^/g and 32.27%, respectively. Compared to free PPI, the EAI of the PPI–OP conjugates achieved an approximate 62.62% increase, while the ESI exhibited a significant elevation of 80.39% (*p* < 0.05). Research has demonstrated that the EAI and ESI of proteins are closely associated with their solubility. Higher protein solubility promotes the distribution of proteins in the aqueous phase, enabling rapid migration to the oil–water interface [31]. The interfacial adsorption state in mixtures may be affected by external factors, including temperature and ionic strength, during the emulsification process due to the relatively weak non-covalent bond interactions. In contrast, covalent bonds have higher bond energies and more stable structures, leading to minimal changes in molecular shape and structure during emulsification. Additionally, glycosylation could alter proteins’ tertiary and quaternary structure, thereby affecting emulsifying performance [31]. During the covalent binding of proteins with polysaccharides, the protein expanded and exposed many hydrophobic groups, increasing its lipophilicity. Simultaneously, the hydrophilic groups of polysaccharides further enhanced the hydrophilicity of the PPI–OP conjugate. Thus, when preparing water–oil systems based on protein–polysaccharide complexes, the amphiphilic proteins first adsorbed rapidly at the oil–water interface, forming a viscoelastic film. The hydrophilic polysaccharides then further strengthened the interface, preventing oil leakage and droplet aggregation [39]. Additionally, polysaccharides provided a stable spatial steric hindrance to emulsion droplets due to their thickening and gelling properties in the aqueous phase. This synergistic effect enables the PPI–OP complex to form a more robust adsorption layer at the interface, leading to the stable encapsulation of oil droplets with enhanced stability. Therefore, PPI–OP conjugates exhibited excellent emulsifying performance.

### 3.3. Microstructure of Pickering Emulsions

**Figure 4:**
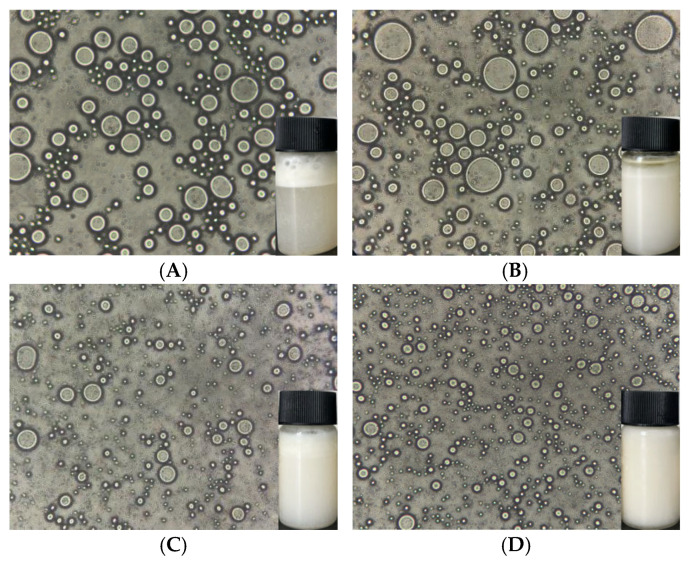
The optical microscopy image (40×) of Pickering emulsions stabilized by (**A**) PPI; (**B**) OP; (**C**) PPI–OP mixtures; and (**D**) PPI–OP conjugates.

The oil droplet sizes in emulsions stabilized by different kinds of nanoparticles showed obvious variations. As shown in Figure 4, all the emulsion droplets were uniformly distributed, spherical in shape, and had distinct boundaries. The droplet size reduced after mixing PPI and OP, and this reduction was even more pronounced following the Maillard reaction coupling. This phenomenon can be attributed to structural and interactional modifications induced by the Maillard reaction, which enhanced the adsorption and stabilization abilities of PPI and OP at the interface. Smaller droplets possess a larger interfacial area, providing more sites for protein adsorption, effectively reducing the droplet size and improving emulsion stability [40]. Additionally, the interfacial film formed by the conjugates on the droplet surfaces helped maintain their smaller size and prevented aggregation and growth induced by collisions during storage. These findings align with the results of the particle size analysis.

**Figure 5:**
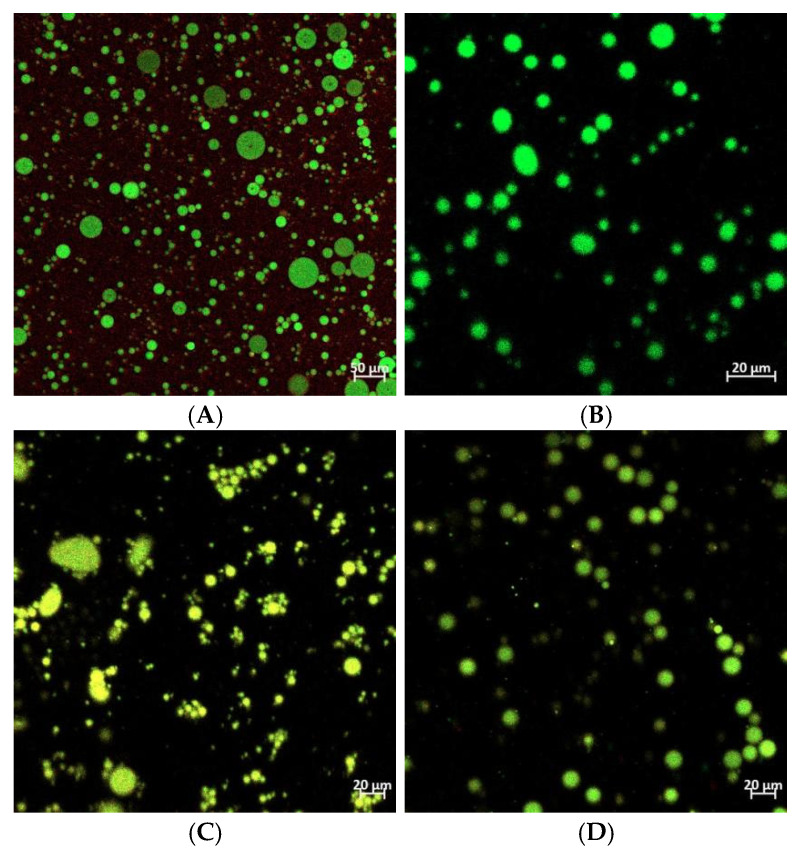
The CLSM image of Pickering emulsions stabilized by (**A**) PPI; (**B**) OP; (**C**) PPI–OP mixtures; and (**D**) PPI–OP conjugates.

Laser confocal microscopy can be used to observe the distribution of the complex in the oil–water system intuitively. As shown in Figure 5, the fluorescently labeled oil droplets appeared green, while the proteins appeared red. In the emulsion stabilized by free PPI (Figure 5A), most of the proteins remained in a dispersed state in the aqueous phase. In contrast, as shown in the mixture and conjugate emulsions (Figure 5C,D), the PPI–OP complexes adhered to the surface of the droplets, forming a more closely packed layer around them, with fewer identifiable free complexes. Previous studies have shown that protein–polysaccharide complexes are capable of forming viscoelastic interfacial films, preventing oil droplet aggregation and thereby stabilizing the emulsion to a certain extent [41]. However, in the PPI–OP mixed emulsion, changes in the droplet shape were observed, with a relatively disordered distribution, and the presence of some larger droplets was also observed (Figure 5C). This may be due to the limited stability of the non-covalent binding between PPI and OP. Additionally, the larger particle size partially masks the exposed hydrophobic residues, reducing surface hydrophobicity and facilitating its effortless desorption from the oil–water interface, thus triggering droplet bridging and aggregation [42]. This indicated that the adsorption of PPI and OP in the mixed emulsion was relatively unstable. In contrast, Maillard conjugates were more effective in reducing oil–water interfacial tension and formed a more stable and compact interfacial adsorption layer due to their better emulsifying properties. This resulted in emulsion droplets stabilized by PPI–OP conjugates being well dispersed, independent of each other, and dispersed more finely and uniformly in the aqueous phase.

### 3.4. Emulsion Stability Evaluation Against Environmental Stresses

#### 3.4.1. PH Stability of Emulsions

**Figure 6:**
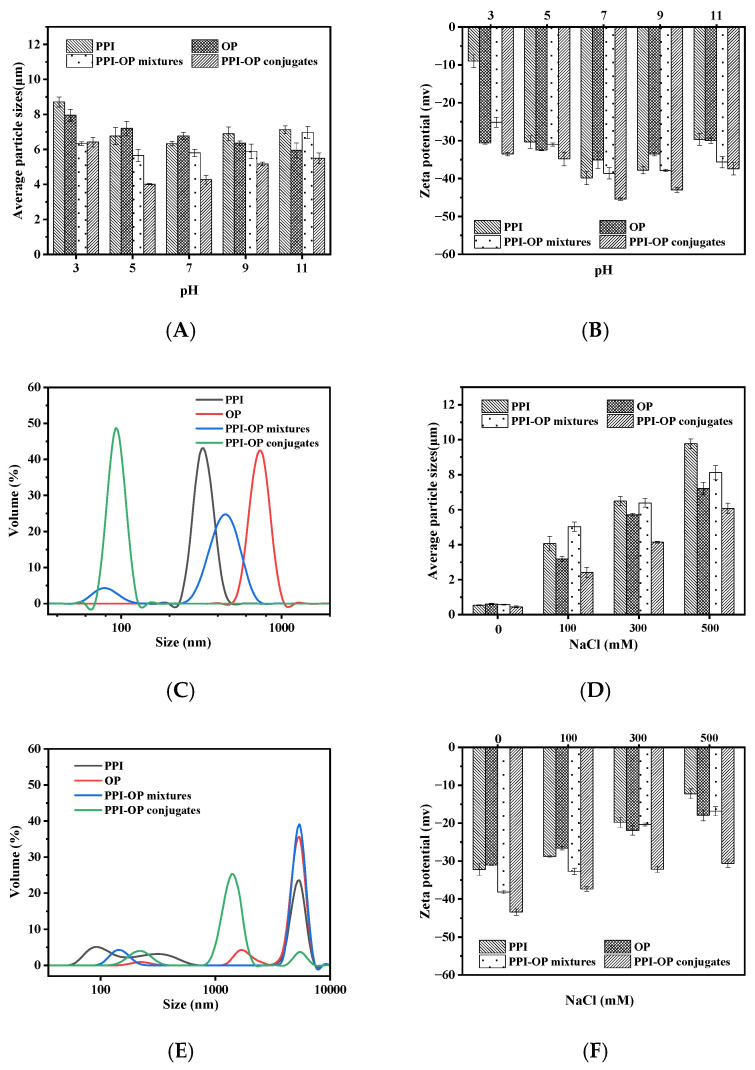
Average particle sizes (**A**,**D**) and ζ—potentials (**B**,**F**) of PEs stabilized by PPI, OP, PPI–OP mixtures, and PPI–OP conjugates at different pH levels and different concentrations of NaCl. The size distributions of PEs at pH 3 (**C**) and 500 mM NaCl (**E**), respectively, as determined using DLS.

The stability of emulsions stabilized by PPI, OP, PPI–OP mixtures, and PPI–OP conjugates under different pH conditions exhibited distinct trends. In the PPI group, the particle size first decreased and then increased as pH changed, reaching its smallest value (6.33 μm) at a pH of 7 (Figure 6A). The changes in absolute charge values followed a similar trend, peaking at −39.85 mv at a pH of 7 (Figure 6B). This indicated that PPI can only provide good emulsion stability near neutral pH values. However, under highly acidic (pH = 3) and highly alkaline (pH = 11) conditions, the weak physical adsorption forces (electrostatic adsorption and van der Waals forces) responsible for PPI’s emulsifying properties were easily disrupted, leading to an imbalance between adsorption and repulsion forces and subsequent emulsion destabilization [43]. In the OP group, the particle size gradually decreased as pH increased, while absolute charge values remained relatively stable. The large particle size was observed at a pH of 3 (Figure 6C), likely due to the protonation of acidic groups, such as carboxyl groups on polysaccharide molecules in an acidic environment. This protonation may have altered the conformation of the polysaccharide molecular chains, reducing their extension and thereby weakening their stabilizing effect on emulsion droplets and promoting aggregation [44]. At a neutral pH, the OP maintained good solubility and molecular conformation and formed a uniform, stable adsorption layer around the emulsion droplets, effectively preventing aggregation. At a pH of 11, the smallest particle size was recorded (5.95 μm). This may be due to the deprotonation of acidic groups, leading to molecular chain extension [45]. This enhanced electrostatic repulsion and steric hindrance, reducing particle size. However, excessive alkalinity could degrade polysaccharide structures, leading to increased particle size and reduced emulsion stability. For PPI–OP mixtures, the overall trend resembled that of the PPI group. However, under all pH conditions, except pH = 11, the particle size was smaller, and the absolute charge was higher than that of the PPI. This suggests that the added polysaccharides provided steric hindrance and enhanced electrostatic repulsion. The thick-layer structure of polysaccharide molecules increased the spatial distance between particles, and the charges of polysaccharides enhanced the electrostatic repulsion force, leading to a reduction in particle size and increased absolute potential values [24]. The PPI–OP mixtures formed an interfacial layer on the surface of the emulsion droplets under alkaline conditions due to the change in charge distribution of the interfacial layer. On one hand, the further dissociation of acidic groups on proteins and polysaccharides increased the negative charge density. However, on the other hand, excessive charge accumulation could alter ionic strength, disrupting the balance of electrostatic repulsion and promoting droplet aggregation, ultimately increasing particle size. The PPI–OP conjugates exhibited significantly lower particle sizes and greater absolute charge value (>30 mv) across the pH range of 3–11. This indicated that the conjugates effectively resisted acidic and alkaline destabilization. The covalent bonding between OP and PPI created a stable structure, which prevented particle aggregation under different pH conditions. Additionally, some functional groups were fixed on the surface of the particles through covalent coupling, which allowed for a stable charge distribution under different pH conditions, sustaining strong inter-molecular electrostatic repulsion. In addition, the coupling process may have changed the original charge distribution of proteins and polysaccharides, exposing more acidic groups on particle surfaces and increasing the negative charge density, thereby enhancing the physical stability of the emulsion under different pH conditions.

#### 3.4.2. Ionic Stability of Emulsions

In the PPI group, the droplet size of the oil gradually increased with NaCl concentration (Figure 6D). At 500 mM of NaCl, the particle size rapidly increased to 9.77 μm due to the salt-bridge formation, leading to emulsion denaturation and aggregation. The presence of NaCl weakened the electrostatic repulsion between nanoparticles, prompting aggregation. After adding the NaCl, particle size distribution in emulsion stabilized by PPI showed a multi-peak pattern (Figure 6E). Moreover, the particle size increased, and the distribution broadened with the change in ionic strength. Regarding zeta potential, its absolute value decreased with increasing NaCl concentration (Figure 6F), which indicated compression of the electric double layer on particle surfaces, making it thinner and reducing the surface potential layer. This may be the reason for PPI poor emulsion stability in a high-salt ion environment. The OP group showed a similar trend to the PPI group. As the NaCl concentration increased, salt ions disturbed inter-molecular interactions, enlarged droplet size, and broadened the particle size distribution. Meanwhile, the absolute value of the potential decreased due to weakened electrostatic repulsion between particles, and emulsion stability was reduced. Compared to the PPI group, the PPI–OP mixture group exhibited relatively smaller particle sizes at 300 and 500 mM NaCl, indicating that OP’s steric hindrance and electrostatic effects inhibited the aggregation. The particle size distribution in the PPI–OP mixture group was also narrower than that in the PPI group, which indicated relatively better stability. Similarly, the absolute value of the potential was slightly higher than that of the PPI group. Since the charges carried by OP increased the electrostatic repulsion of the system, it was conducive to maintaining the stability of the emulsion. However, the potential still significantly decreased with increasing NaCl concentration. In the PPI–OP conjugate group, the particle size was significantly smaller (*p* < 0.05), and its growth rate was relatively slower compared with other groups, demonstrating enhancement in anti-coalescence performance. The narrower particle size distribution indicated superior stability. Covalent bonding between PPI and OP enabled the droplets to effectively maintain electrostatic repulsion in a high salt-ion environment, accounting for the significantly reduced particle aggregation. Therefore, the emulsions stabilized by PPI–OP conjugates showed good stability and resistance to salt-induced destabilization.

### 3.5. Stability of Emulsions to Lipid Oxidation

**Figure 7:**
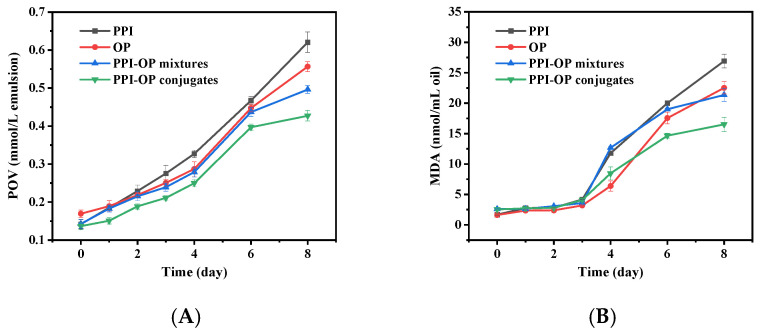
The peroxide value (**A**) and amount of malondialdehyde (**B**) of the PEs stabilized by PPI, OP, PPI–OP mixture, and PPI–OP conjugate. Stored at 25 °C for 8 days.

The stability of emulsion against lipid oxidation was evaluated over eight days at 25 °C by measuring the concentrations of primary (lipid hydroperoxides) and secondary (MDA) reaction oxidation products (Figure 7). The peroxide value (POV) of the emulsion increased moderately during the first four days and then sharply rose from days four to eight. However, POV in the PPI–OP conjugate group remained significantly lower than in other groups. On the eighth day, the POV in PPI–OP conjugates was merely 68.64%, 76.77%, and 86.08% of the POV in PPI, OP, and PPI–OP mixture groups, respectively. Additionally, the MDA value remained stable within the first three days but showed a significant difference on the fourth day. The PPI–OP conjugate-stabilized emulsion significantly inhibited the rapid increase in the MDA value, while the MDA value in the OP group grew faster than that in the PPI–OP conjugates with the extension of time. The above results showed that lipid oxidation was relatively inhibited throughout the storage process in PPI–OP conjugate groups. This may be because the conjugates formed a relatively dense interfacial film on the surface of the oil, which prevented the direct contact between oxygen and oil, reducing the diffusion of oxygen into the interior of the oil. In addition, studies have shown that, during the Maillard reaction, a variety of substances with antioxidant activity were formed, such as pyrrole, pyridine, pyrazine, and melanoidins. These substances have excellent antioxidant properties and can scavenge free radicals and inhibit lipid oxidation through different mechanisms [46].

### 3.6. Interfacial Structure Analysis of Emulsions

**Figure 8:**
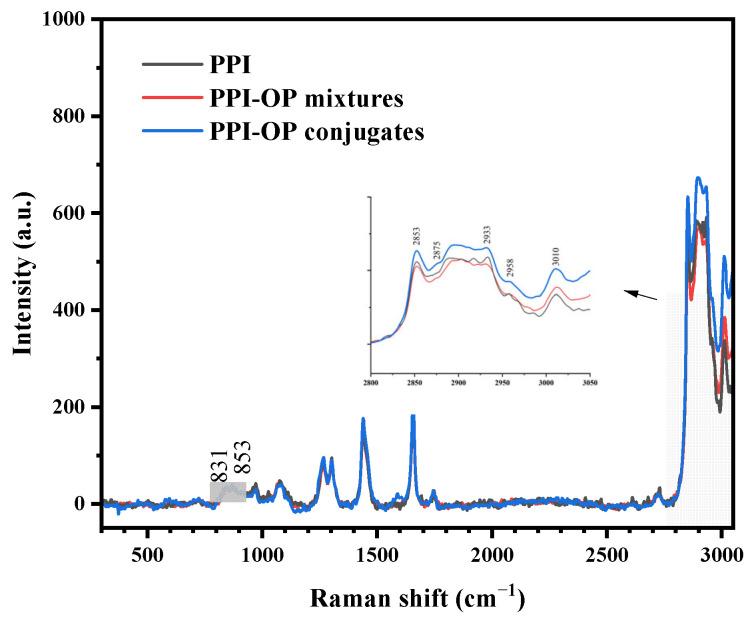
The Raman spectra of Pickering emulsions stabilized by PPI, PPI–OP mixtures, and PPI–OP conjugates.

To investigate the interfacial structural characteristics of emulsion stabilized by the PPI–OP conjugate, Raman spectroscopy was employed to examine the structural features of lipid molecules and their interactions with the interfacial complex. Figure 8 and Table 3 show the Raman spectra of emulsions stabilized by different nanomaterials and the corresponding calculation results. The relative intensity ratio (I_853_/I_831_) near 853 and 831 cm^−1^ reflects the changes in the microenvironment of aromatic amino acids such as tyrosine and phenylalanine residues. This ratio serves as a reliable indicator of the hydrogen bonding involving phenolic hydroxyl groups. The I_853_/I_831_ ratio of the PPI–OP conjugate emulsion further increased to 1.954, indicating that more aromatic amino acids were exposed to the polar environment. These amino acids act as hydrogen bond acceptors on the protein surface, enhancing interactions between the protein and water molecules. The peaks at 2853, 2875, 2933, 2958, and 3010 cm^−1^ corresponded to the symmetric stretching vibrations of –CH_2_ and –CH_3_, the asymmetric stretching of –CH_2_ and –CH_3_, and the symmetric stretching of the cis double bond =C–H, respectively (Figure 8). The noticeable broadening of the spectral pattern in the conjugate emulsion is usually attributed to the decrease in the ordered conformation of the lipid acyl chains and an increase in their dynamics. The I_2853_/I_2875_ ratio reflected interactions between the carbon chains. A lower ratio indicates stronger interactions between the interfacial layer materials and the lipids [47]. Meanwhile, the I_2933_/I_2875_ ratio represented the influence of trans/side chain isomerization among carbon chains. An increase in peak intensity at 2875 cm^−1^ indicated enhanced symmetric stretching of side chain –CH_3_, leading to a decreased ratio, and further reflected increased chain interactions. These significant changes indicated the insertion of protein or polysaccharide chains into the acyl chains of the oil and altered the lipid–protein interactions. Previous studies have demonstrated a strong positive correlation between emulsion stability and lipid chain disorder [48]. The PPI–OP covalent conjugate exposed more hydrophobic side chains within the oil phase. Additionally, inserting PPI and OP chains into the oil’s acyl chains enhanced the interaction between lipids and protein–polysaccharide complexes, thereby improving emulsion stability. These structural alterations provided further evidence that emulsion stability has a close association with the development of a thick and compact interfacial layer on the oil droplet surface.

### 3.7. Rheological Analysis

**Figure 9:**
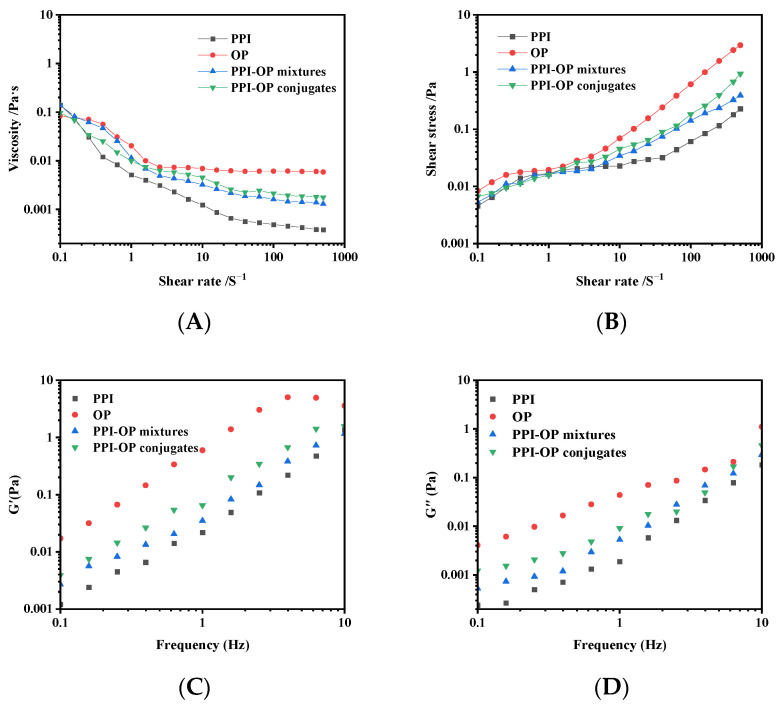
Emulsion viscosity as a function of shear rate (**A**), shear stress as a function of shear rate (**B**), storage modulus (G′) (**C**), and loss modulus (G″) (**D**) as a function of frequency of different Pickering emulsions.

The rheological behavior of the prepared emulsions was characterized using a rotational rheometer. Figure 9 illustrates the variation of emulsion viscosity with respect to shear rate, as well as the alteration in G′ and G″ during frequency scanning.

The apparent viscosities in all emulsions gradually decreased with the increasing shear rate (Figure 9A), conforming to the behavior of non-Newtonian fluids characterized by shear-thinning and pseudoplasticity [49]. As shown in Figure 9B, the emulsion prepared from the PPI–OP conjugate exhibited a higher shear stress value. The emulsions stabilized by free PPI were unstable and easily delaminated under low shear forces, exhibiting a relatively low apparent viscosity. In contrast, OP had a relatively high viscosity, and its covalent modification with PPI increased the apparent viscosity of the protein-based emulsion. Research has shown that the covalent conjugation of polysaccharides can change the charge distribution of conjugates [50]. The absolute zeta-potential of PPI–OP conjugates significantly increased to 44.39 mV, indicating that they could enhance electrostatic repulsion within the emulsion system. According to previous studies, the dispersion state of polymer chains is maintained by electrostatic repulsion, effectively preventing chain aggregation. Meanwhile, covalent linkages between protein and polysaccharide molecules establish a stable interconnected network, while localized hydrophobic interactions and hydrogen bonding synergistically promote the formation of high-density crosslinking nodes [47,51]. These combined forces contribute to a dense and homogeneous network structure within the covalent conjugates, which restricts oil droplet mobility and thereby enhances the apparent viscosity of the emulsion [52,53]. The formation of such a network-like membrane structure can significantly improve emulsion stability by suppressing droplet aggregation [54]. The observed increases in shear stress and viscosity in our research suggest the formation of a more densely crosslinked network structure, as increased viscoelasticity is widely recognized to correlate with higher interfacial packing density [55]. This structural reinforcement effectively restricts droplet mobility, thereby reducing coalescence and improving emulsion stability.

The frequency sweep curve (Figure 9C,D) showed that the emulsion storage modulus G′ and loss modulus G″ increased significantly with the range of 0.1–10 Hz. Among them, G′ was slightly higher than G″, and the loss tangent (tanδ < 1) indicated that elasticity dominated the formation of the emulsion gel structure, giving the emulsion typical solid elastic characteristics [56]. In addition, the emulsion stabilized by PPI–OP conjugates demonstrated significantly enhanced viscoelasticity, with both the storage modulus and loss modulus markedly higher than those of PPI or PPI–OP mixture stabilized systems. This suggested that the incorporation of polysaccharide molecules and covalent interactions contributed to forming a denser gel structure and improved the emulsion gel’s mechanical properties, enhancing the resistance of the emulsion droplets to the external environment [57].

### 3.8. Encapsulation Efficiency of API in Emulsion

The encapsulation efficiency of nutrients is directly related to the ability of emulsion to encapsulate and protect them and serves as a crucial indicator for assessing emulsion performance. Among the emulsions, the prepared PPI–OP conjugates exhibited the highest encapsulation efficiency (91.52 ± 0.75%). Compared to the encapsulation efficiency of PPI and PPI–OP mixtures, this represents an increase of 17.98% and 8.14%, respectively (*p* < 0.05). The modification of PPI by polysaccharides containing various functional groups may increase the covalent binding sites for the encapsulated substances [58], thus embedding more lipids. On the other hand, the hydroxyl groups on the sugar unit of polysaccharide and glycosidic oxygen bonds can form hydrogen bonds with the hydroxyl groups of active substances, while hydrophobic regions of polysaccharides simultaneously engage with the nonpolar domains of the active substances [47]. These spatially and energetically complementary non-covalent interactions synergistically enhance molecular binding affinity, thereby significantly improving encapsulation efficiency. As a result, the encapsulated substances can be more firmly encapsulated inside, thereby reducing their leakage and increasing the encapsulation efficiency. In addition, compared to non-covalent encapsulation, emulsion stabilized with PPI–OP conjugates demonstrated higher stability and better resistance to environmental changes, thereby maintaining its integrity better and reducing the release of encapsulated substances.

### 3.9. Stability of API in Emulsion

**Figure 10:**
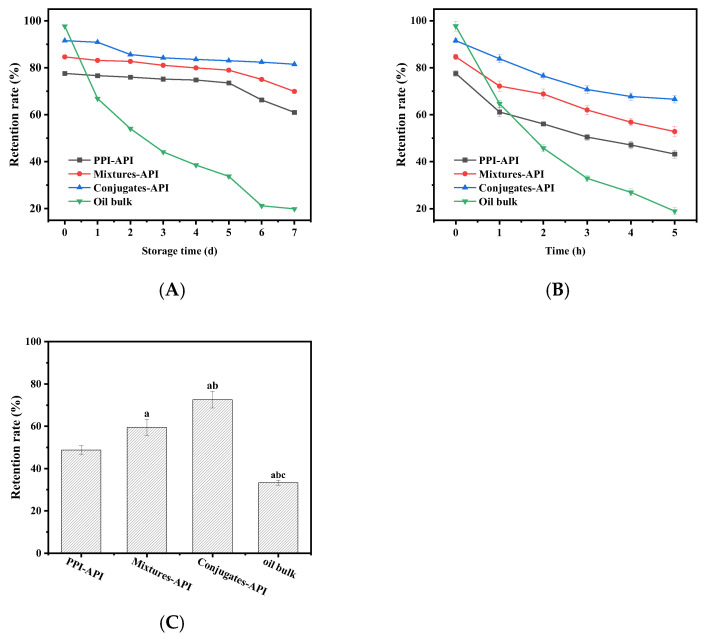
The retention rate of API in PPI-API, Mixtures-API, and Conjugates-API emulsion delivery systems and oil bulk storage at 25 °C (**A**), heated at 95 °C (**B**), and 5 h ultraviolet irradiation (**C**). “a”, “b”, and “c” represent a significant difference (*p* < 0.05) vs. the PPI-API group, the Mixtures-API group, and the Conjugates-API group, respectively.

To assess the protective effect of the emulsions stabilized by the PPI–OP conjugate on apigenin (API) against environmental stress, the influences of storage time, heat treatment (95 °C), and UV radiation on the retention rate of API were evaluated (Figure 10). After 7 days of storage at 25 °C, the free API group exhibited a drastic API loss of 79.66%, whereas the PPI-API, Mixture-API, and Conjugates-API groups retained significantly higher API levels, with losses of only 21.36%, 17.38%, and 10.96%, respectively. This retention advantage in Conjugates-API emulsion is attributed to its enhanced stability, which suppresses API migration from the oil phase to the aqueous phase, thereby minimizing exposure to pro-oxidative factors. Similarly, under accelerated thermal stress (95 °C for 5 h), the free API group suffered an 80.61% loss, which was primarily attributed to the thermal degradation of its flavonoid backbone (cleavage of phenolic hydroxyl groups and conjugated double bonds) [56]. In contrast, API retention losses in PPI-API, Mixture-API, and Conjugates-API groups were significantly lower at 44.27%, 37.58%, and 27.24%, respectively. The superior thermal stability of emulsion stabilized by the PPI–OP conjugate arises from the covalent bonding between protein and polysaccharide moieties, which resists heat-induced interfacial rupture, thereby preserving API encapsulation efficiency. Under UV radiation (Figure 10C), the free API control exhibited severe degradation, with retention plummeting to 33.32 ± 1.18%. In contrast, the PPI-API and Mixtures-API groups demonstrated improved stability, retaining 48.82 ± 2.10% and 59.47 ± 3.87% of API, respectively. The enhanced protection in physical mixtures correlates with the radical scavenging activity of polysaccharides [17]. Notably, the covalent conjugate system achieved superior API retention (72.60 ± 3.91%, Δ = 118.3% vs. free API). These results demonstrated that the emulsion stabilized by PPI–OP conjugates more effectively inhibited API degradation under UV radiation, exhibiting superior protective efficacy.

### 3.10. Effect of Emulsion Embedding on the Digestibility of Api In Vitro

**Figure 11:**
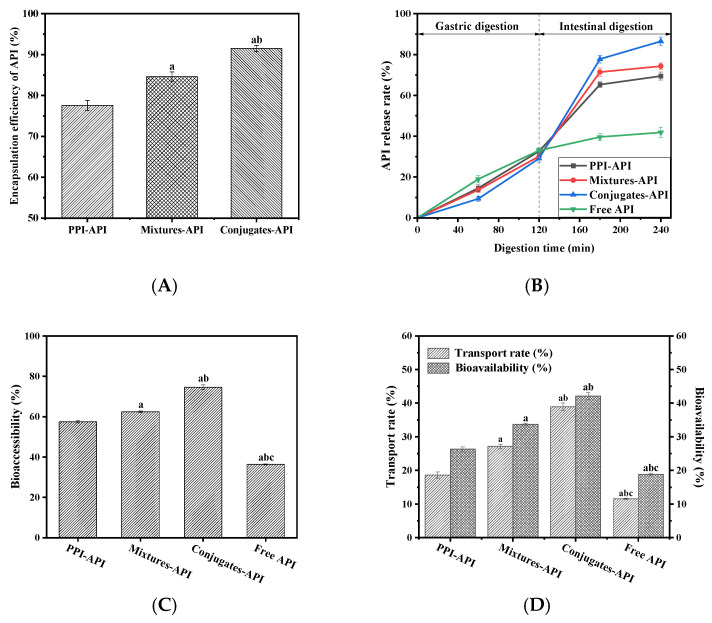
Encapsulation efficiency (%) (**A**), Apigenin release rate (%) (**B**), bioaccessibility (%) (**C**), transport rate (%), and bioavailability (%) (**D**) of PPI-API, Mixtures-API, and Conjugates-API emulsion delivery systems. “a”, “b”, and “c” represent a significant difference (*p* < 0.05) vs. the PPI-API group, the Mixtures-API group, and the Conjugates-API group, respectively.

In vitro simulated digestion was used to conduct a simulation study on the gastrointestinal digestion process of apigenin under different carriers to evaluate the release rate and bioavailability of apigenin. As shown in Figure 11, the free API group exhibited a relatively high release rate, while the PPI–OP conjugate emulsion delivery system significantly protected API in the stomach. This suggested that the structure of the PPI–OP conjugate provided better resistance to pepsin hydrolysis and higher pH fluctuations, while most of the API remained intact within the emulsion. The release of API in intestinal digestive fluid occurred in 120–240 min. The free API group maintained a relatively low final release rate of 41.84 ± 2.38%, likely due to its strong hydrophobicity, which reduced its solubility in the aqueous intestinal environment, leading to poor absorption. Moreover, it had insufficient stability in the acidic and alkaline environment of the gastrointestinal tract and may undergo degradation or structural transformation. In contrast, the release rate of API in the emulsion stabilized by the PPI–OP conjugate significantly increased in the intestinal digestive stage (*p* < 0.05), indicating sustained controlled release with slower degradation. This behavior contrasted with the rapid metabolism and decomposition observed in free API. Among these, the API release rate in the PPI–OP conjugate emulsion group reached a maximum of 86.49 ± 2.04%. As illustrated in Figure 11, API encapsulated in emulsion has significantly improved bioaccessibility compared to free API. The bioaccessibility of API encapsulated in Pickering emulsions stabilized by PPI, PPI–OP mixtures, and PPI–OP conjugates were 57.47 ± 0.57%, 62.41 ± 0.41%, and 74.58 ± 1.19% respectively. The interfacial layer formed by the Maillard conjugate on the droplet surface and the smaller emulsion particle size contributed to a 105.28% increase in bioaccessibility compared to free API. These results suggest that the emulsion stabilized by the PPI–OP Maillard conjugate has the potential as an intestine-responsive release system for bioactive substances.

### 3.11. Caco-2 Cellular Absorption and Transmembrane Transport Efficiency of API with Different Encapsulation Methods

Firstly, the cytotoxicity of in vitro simulated intestinal digests on Caco-2 cells was studied. As shown in Figure S1, when the intestinal digests were diluted 100-fold with the cell culture medium, the survival rates of Caco-2 cells in all groups were greater than 90%, indicating no obvious toxic effect on the cells. Therefore, a maximum dilution ratio of 1:100 was finally selected for subsequent cell studies.

In research on bioactive substances, their transport rates and uptake rates in Caco-2 cells are key indicators for measuring efficacy. Compared to free API, API encapsulated within emulsions exhibited higher transport rates and bioavailability (*p* < 0.05) (Figure 11). Protein-stabilized emulsions usually exhibited higher cellular uptake than traditional emulsions because the exposed protein ligands may be recognized by membrane receptors, promoting their cellular internalization [59]. In addition, API has a low solubility in aqueous solutions, limiting its ability to interact with the hydrophilic cell surface, thus affecting its cellular interaction and transmembrane transport and, consequently, reducing the cellular uptake rate. However, compared with PPI-API and Mixtures-API emulsions, the Conjugates-API emulsion demonstrated greater transport and absorption. Studies have demonstrated that, in free protein emulsions, free amino acid residues on the protein surface bind to cell receptors via nonspecific weak interactions (e.g., electrostatic attractions and hydrophobic interactions). Unlike conjugate-stabilized emulsion, these non-directed associations fail to establish ordered supramolecular architectures, exhibit conformational rigidity, and demonstrate transient binding with rapid dissociation kinetics. This results in low binding specificity and suboptimal affinity [60,61]. Furthermore, the inherent characteristics of proteins—their bulky molecular size and high surface hydrophilicity—fundamentally restrict passive transmembrane permeability through biological membranes [61]. In emulsions stabilized by PPI–OP mixtures, the absence of covalent conjugation between PPI and OP precludes the formation of a cohesive network. The dominant non-covalent interactions yield structurally labile interfaces, leading to compromised protective efficacy for encapsulated API, including diminished oxidative stability and accelerated payload leakage [62]. In contrast, the polysaccharide conjugation induced conformational rearrangements in the protein structure, facilitating the exposure of its functional groups. This structural remodeling enabled cooperative hydrogen bonding and hydrophobic interactions with target receptors, thereby enhancing receptor binding specificity and affinity [51]. Additionally, polysaccharides can penetrate Caco-2 cells, primarily via endocytosis-mediated pathways [63]. Both of the above reasons effectively enhance cellular endocytic activity and significantly improve the cell uptake rate of API. These results demonstrate that the PPI–OP covalent conjugate significantly improved emulsion stability and boosted API transport efficiency, while the low ζ-potential value of the conjugate emulsion may contribute to improved internalization [64]. Additionally, the results of simulated digestion showed that the emulsion stabilized by the PPI–OP conjugate could achieve the controlled release of API, with gastric protection and intestinal-targeted delivery to enhance bioavailability. This continuously provides raw materials for cell uptake, significantly improving the uptake efficiency of API.

## 4. Conclusions

This research explored the physicochemical properties of PPI and OP conjugates formed via the Maillard reaction, focusing on their potential as Pickering stabilizers and their role in API delivery using emulsions. The Maillard reaction was performed under optimized conditions (70 °C, pH of 10, and 12 h) with a 1:1 mass ratio to generate pea protein isolate–okra flower polysaccharide conjugate with the highest grafting degree of 22.80 ± 0.26%. Infrared analysis verified the covalent binding of PPI with OP. The Maillard reaction brought structural alterations in PPI, including transformations in the secondary structure, depletion of intrinsic fluorescence, and modifications of microstructure. Additionally, the introduction of the OP reduced the turbidity and particle size and increased the absolute potential values of PPI. The above alterations in the PPI structure significantly improved emulsifying activity and emulsion stability. The emulsion stabilized by conjugates possessed stronger stability than those of PPI, OP, and PPI–OP mixtures under different environmental stresses. This can be attributed to the PPI–OP conjugate inducing an interface adsorption layer and stable steric hindrance, which increased intermolecular electrostatic repulsions and reinforced lipid–protein interactions. Additionally, the PPI–OP conjugate enhanced apparent viscosity and viscoelastic properties of emulsion via covalent cross-linking, improving emulsion stability. Moreover, conjugate-stabilized emulsions also exhibited a higher encapsulation efficiency of 91.52% and superior protective efficacy of API compared to other emulsions. They could also control the release of Apigenin in simulated gastrointestinal fluids and improve bioaccessibility, as well as the enhancement of absorption and transmembrane transport efficiency of API in caco-2 cells. In summary, PPI–OP conjugates can be regarded as promising emulsifiers for the development of stabilizing food emulsions with excellent functional properties and present great potential applications in delivering lipid-soluble nutrients.

## Figures and Tables

**Table 1:** Secondary structure content of PPI, PPI–OP mixtures, and PPI–OP conjugates.

Sample	α-Helix (%)	β-Sheet (%)	β-Turn (%)	Random Coil (%)
PPI	26.2 ± 0.04	31.3 ± 0.11	10.4 ± 0.04	32.1 ± 0.09
PPI–OP mixtures	17.0 ± 0.03 ^a^	29.1 ± 0.12 ^a^	14.2 ± 0.06 ^a^	39.7 ± 0.11 ^a^
PPI–OP conjugates	11.9 ± 0.04 ^ab^	27.8 ± 0.10 ^ab^	16.7 ± 0.08 ^ab^	43.6 ± 0.12 ^ab^

Note: “a”, “b” represent a significant difference (*p* < 0.05) vs. the PPI group and the PPI–OP mixtures group, respectively.

**Table 2:** Turbidity, particle size, and zeta-potential of PPI, PPI–OP mixtures, and PPI–OP conjugates.

Sample	Turbidity	Particle Size (nm)	Zeta-Potential (mV)
PPI	1.93 ± 0.01	535.70 ± 0.83	−30.37 ± 2.33
OP	1.01 ± 0.01 ^a^	607.75 ± 1.28 ^a^	−32.50 ± 0.10
PPI–OP mixtures	1.86 ± 0.03 ^b^	288.15 ± 1.06 ^ab^	−38.18 ± 0.45 ^ab^
PPI–OP conjugates	1.26 ± 0.02 ^abc^	212.05 ± 0.64 ^abc^	−44.39 ± 1.53 ^abc^

Note: “a”, “b”, and “c” represent a significant difference (*p* < 0.05) vs. the PPI group, the OP group, and the PPI–OP mixtures group, respectively.

**Table 3:** Relative intensity ratio of the tyrosine bimodal spectral band (at 853/831 cm^−1^), principal carbon chains (at 2851/2892 cm^−1^), and the overlap of trans/deflected isomerization between carbon chains (at 2930/2892 cm^−1^) of PPI, PPI–OP mixtures, and PPI–OP conjugates.

Sample	*I*_853_/*I*_831_	*I*_2853_/*I*_2875_	*I*_2933_/*I*_2875_
PPI	0.927 ± 0.004	1.207 ± 0.008	1.229 ± 0.010
PPI–OP mixtures	1.501 ± 0.005 ^a^	1.168 ± 0.004 ^a^	1.196 ± 0.001 ^a^
PPI–OP conjugates	1.954 ± 0.012 ^ab^	1.155 ± 0.003 ^ab^	1.187 ± 0.002 ^ab^

Note: “a” and “b” indicate a significant difference (*p* < 0.05) vs. the PPI group and the PPI–OP mixtures group, respectively.

## Data Availability

The original contributions presented in the study are included in the article/Supplementary Material, further inquiries can be directed to the corresponding authors.

## Supplementary Materials

The following supporting information can be downloaded at: https://www.mdpi.com/article/10.3390/foods14111923/s1, Table S1: Inorganic salt composition for simulated digestive solution in vitro; Figure S1: The effects of intestinal digesta with different dilution ratios on the viability of Caco-2 cells, which are PPI-API (A), Mixtures-API (B), Conjugates-API (C), and free API (D) respectively; Figure S2: Effects of temperature (A), mass ratio (B), pH (C) and heating time (D) on the grafting degree and browning degree of PPI-OP conjugates.
