# Supplementary material for: Okra Flower Polysaccharide–Pea Protein Conjugates Stabilized Pickering Emulsion Enhances Apigenin Stability, Bioaccessibility, and Intestinal Absorption In Vitro
Source: Foods. 2025 May 28;14(11):1923. doi: 10.3390/foods14111923 (PMC12154403; doi:10.3390/foods14111923)
Supplement: Supplementary file 1 [file foods-14-01923-s001.zip › foods-3645068-supplementary.pdf]

Table S1. Inorganic salt composition for simulated digestive solution *in vitro*

| Reagent name | SGF(mM) | SIF (mM) |
|---|---|---|
| KCl | 6.9 | 6.8 |
| $KH_2PO_4$ | 0.9 | 0.8 |
| $NaHCO_3$ | 25 | 85 |
| NaCl | 47.2 | 38.4 |
| $MgCl_2(H2O)_6$ | 0.12 | 0.33 |
| $(NH4)_2CO_3$ | 0.5 | - |
| $CaCl_2(H_2O)_2$ | 0.15 | 0.6 |

Note: mM represented the final salt concentration contained in each simulated digestive fluid (mmol/mL); "-" indicated that there was no such component.

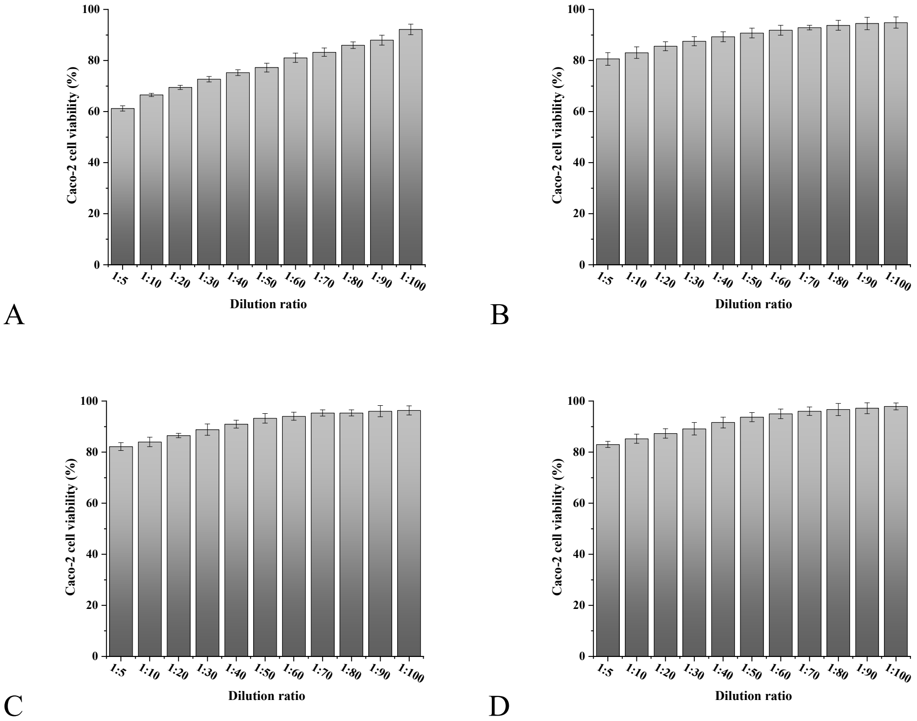


Figure. S1. The effects of intestinal digesta with different dilution ratios on the viability of Caco-2 cells, which are PPI-API (A), Mixtures-API (B), Conjugates-API (C), and free API (D) respectively.

**Determination of grafting and browning degree of PPI-OP conjugates**

A 1% (w/w) pea protein isolate (PPI) and a 1% (w/w) okra flower polysaccharide (OP) solution were mixed at different ratios (4:1, 2:1, 1:1, 1:2, and 1:4 w/w). The pH of each mixture was adjusted to 7, 8, 9, 10, 11, and 12, respectively. The reaction

mixtures were then heated at different temperatures (50, 60, 70, 80 and 90 °C) for different durations (4, 8, 12, 16, 20 and 24 hours) to promote the formation of PPI-OP complexes via the Maillard reaction. After thermal treatment, the reaction was terminated by cooling the solutions in an ice bath for 5 minutes. Each solution was then subjected to dialysis at 4 °C for 24 hours to obtain PPI-OP Maillard conjugates. A portion of each dialyzed solution was preserved by adding 2.5 mg of sodium azide ($NaN_3$) (0.025 %, w/v) and stored at a 4 °C. The remaining portion was freeze-dried for further analyses.

The grafting degree (DG) of the PPI-OP conjugates was evaluated by measuring the reduction in free amino groups after the Maillard reaction using the o-phthaldialdehyde (OPA). Briefly, 200 μL sample solution containing 2.5 mg/mL protein was mixed with 4 mL freshly prepared OPA reagent. The reaction mixture was incubated in a water bath at 35°C for 2 minutes, and then the absorbance was measured immediately at 340 nm. The DG were calculated using the following formula:

$$DG = \frac{A_1 - A_2}{A_1} \times 100 \qquad (1)$$

Where $A_1$ represents the content of free amino groups in the PPI-OP mixture, and $A_2$ represents the content of free amino groups in the covalent complex.

The browning intensity of the Maillard reaction under different reaction conditions, was evaluated by photographing the sample solutions. The degree of browning was qualitatively assessed based on the visual color depth.

**Influence of different preparation conditions on the degree of grafting and browning of PPI-OP conjugates**

As shown in Figure S3, the DG increased with temperature upto a point, then declined at higher temperature (Figure S3A). The DG peaked at 70 °C and 80 °C, reaching 21.94 ± 0.52% and 22.96 ± 0.44% respectively. Browning intensity was also most pronounced at these temperatures, indicating that moderate heating enhanced molecular activity and increased the frequency of effective collisions between protein

and polysaccharide molecules, thereby accelerating the Maillard reaction. However, when the temperature exceeded 80 °C, it likely caused protein denaturation or degradation of polysaccharides, impeding the reaction. With the increase in OP concentration, the DG followed a similar trend of rising and then decreasing (Figure S3B). The highest DG (21.84 ± 0.37%) was observed at a 1:1 mass ratio (w/w) of PPI to OP. Notably, substantial precipitation occurred in all mixtures except the 1:1 ratio group. The above results indicated that the concentration of polysaccharides enhanced the availability of reactive hydroxyl groups, facilitating reactions with protein amino groups. However, excessive PPI levels induce protein aggregation, reducing the availability of grafting sites. Simultaneously, excessive OP levels create steric hindrance, diluting reactive sites and limiting PPI-OP conjugation. In both cases, fewer grafting reactions occur, ultimately decreasing the degree of grafting (DG). Similarly, increasing the pH led to a rise and subsequent fall in the DG (Figure S3C), with the maximum DG (22.80 ± 0.26%) occurring at pH = 10. This was corroborated by the corresponding browning results. Moderate alkalinity favors the exposure and activation of reactive groups such as -OH, $-NH_2$ and -SH groups. Under alkaline conditions, nucleophilic groups ($-NH_2$) in proteins are prone to lose protons and form negative-charge nucleophiles. Meanwhile, electrophilic groups (C=O) in polysaccharides become polarized, facilitating nucleophilic attacks. This enhances the formation of Schiff bases and other Maillard intermediates, which are relatively stable under alkaline conditions and promote subsequent rearrangements. However, strong alkalinity combined with high temperatures intensifies protein denaturation and polysaccharide degradation, leading to reductions in both DG and browning. As shown in Figure S3D, both DG and browning increased with prolonged heating. However, further extension beyond a certain point resulted in a decrease in DG, likely due to degradation of the conjugated products. From above results, the optimal reaction conditions for preparing PPI-OP Maillard conjugates were identified as 70 °C reaction temperature, 1:1 mass ratio of PPI to OP, pH 10, and a reaction time of 12 h. These conditions were used in subsequent experimental analysis.

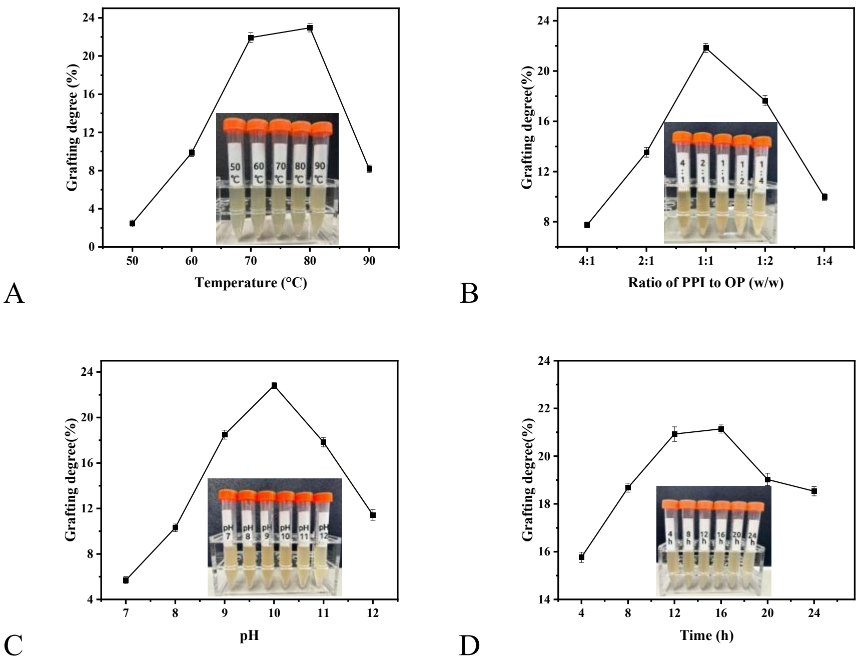


Figure. S2. Effects of temperature (A), mass ratio (B), pH (C) and heating time (D) on the grafting degree and browning degree of PPI-OP conjugates.
